# Opposite effects of GCN5 and PCAF knockdowns on the alternative mechanism of telomere maintenance

**DOI:** 10.18632/oncotarget.15447

**Published:** 2017-02-17

**Authors:** Maya Jeitany, Dalal Bakhos-Douaihy, David C. Silvestre, Jose R. Pineda, Nicolas Ugolin, Angela Moussa, Laurent R. Gauthier, Didier Busso, Marie-Pierre Junier, Hervé Chneiweiss, Sylvie Chevillard, Chantal Desmaze, François D. Boussin

**Affiliations:** ^1^ Laboratoire de Radiopathologie, CEA, Institut de Radiobiologie Cellulaire et Moléculaire, Fontenay-aux-Roses, France; ^2^ INSERM UMR967, Fontenay-aux-Roses, France; ^3^ Université Paris VII, UMR967, Fontenay-aux-Roses, France; ^4^ Université Paris XI, UMR967, Fontenay-aux-Roses, France; ^5^ Laboratoire de Cancérologie Expérimentale, iRCM, DSV, CEA, Fontenay-aux-Roses, France; ^6^ CIGEx, IRCM, Fontenay-aux-Roses, France; ^7^ CNRS UMR8246 Neuroscience Paris Seine-IBPS, Team Glial Plasticity, Paris, France; ^8^ Inserm U1130, Neuroscience Paris Seine-IBPS, Team Glial Plasticity, Paris, France; ^9^ University Pierre and Marie Curie UMCR18, Neuroscience Paris Seine-IBPS, Team Glial Plasticity, Paris, France

**Keywords:** PCAF, GCN5, telomere recombination, ALT

## Abstract

Cancer cells can use a telomerase-independent mechanism, known as alternative lengthening of telomeres (ALT), to elongate their telomeres. General control non-derepressible 5 (GCN5) and P300/CBP-associated factor (PCAF) are two homologous acetyltransferases that are mutually exclusive subunits in SAGA-like complexes. Here, we reveal that down regulation of GCN5 and PCAF had differential effects on some phenotypic characteristics of ALT cells. Our results suggest that GCN5 is present at telomeres and opposes telomere recombination, in contrast to PCAF that may indirectly favour them in ALT cells.

## INTRODUCTION

Telomere elongation capacity has been shown to be one of the prominent features of cancer cells. While telomerase activity is required for most cancer cells, others use a different telomere maintenance mechanism, referred to as alternative lengthening of telomeres (ALT). ALT is thought to be based on homologous recombination (HR)-dependent DNA replication. Some of the main characteristics of ALT cells are the absence of telomerase activity, heterogeneity in telomere length, formation of ALT-associated promyelocytic leukaemia (PML) nuclear bodies (APBs) containing telomeres, and a high frequency of telomere sister chromatid exchanges (T-SCEs) [1].

The shelterin proteins are key players in the homeostasis of chromosome ends that function by capping and thus protecting telomeres and also by modulating the recruitment of telomerase [2]. Shelterin proteins are subject to post-translational modifications, such as phosphorylation, SUMOylation or ubiquitination [3]. These modifications can affect their recruitment to telomeres or their interactions with other proteins or regulate their turnover, resulting in telomeric changes. In ALT cells, formation of APBs can thus be prevented by disrupting the SUMOylation of both the shelterin proteins TRF1 and TRF2 [4]. In telomerase-positive cells, the lysine acetyltransferase general control non-derepressible 5 (GCN5), which is a *bona fide* component of the large multi-subunit complex SAGA (SPT-ADA-GCN5 acetyltransferase), regulates the turnover of TRF1 [5]. GCN5 is required for the association of SAGA with USP22, which is the component of its deubiquitination module, and the subsequent USP22-mediated deubiquitination of TRF1, which inhibits TRF1 degradation by proteasomes, thereby preventing signalling associated with telomere DNA damage and protecting telomeres from fusions.

P300/CBP-associated factor (PCAF) is another lysine acetyltransferase that shares ∼73% amino acid sequence identity with GCN5 [6]. GCN5 and PCAF are mutually exclusive subunits of different SAGA-like or Ada-Two-A-containing (ATAC)-like complexes [7–11]. These complexes contain mutual subunits but are responsible for regulating distinct substrates or targets and thus have different biological roles. PCAF, which possesses an intrinsic ubiquitination activity [12], is involved in many cellular processes, such as transcription, differentiation, proliferation and apoptosis [8, 13]. It modulates the activities of several oncogenes and tumour repressors through acetylation of either histones or transcription factors, consequently impacting cancer progression. In contrast with GCN5, PCAF has not been shown to have a role in telomere maintenance to date.

Down-regulation of *gcn5* has been reported in ALT cell lines [14], whereas upregulation of its homologue *pcaf* expression has been shown to be associated with ALT in mice with lymphoma [15]. Here, we show that the depletion of GCN5 or PCAF in ALT cell lines induces differential effects on some ALT features, which suggests that GCN5 down-regulates ALT through its interactions with USP22, whereas PCAF may indirectly increase telomere recombination in ALT cells.

## RESULTS

### GCN5 but not PCAF interacts with telomeric proteins in both ALT and telomerase-positive cells

We used the human osteosarcoma ALT cell line SAOS-2 and the human ALT glioma stem cells (GSC) line TG20 [16, 17] to investigate the importance of GCN5 and PCAF in ALT cells. These cell lines express *gcn5* and *pcaf* genes at both mRNA and protein levels (Supplementary Figure 1A–1E). Noticeably, PCAF to GCN5 protein ratio was higher in TG20 cells, than in telomerase-positive (non ALT) human GSCs (TG16, TG1N, OB1 and TG10 [17], Supplementary Figure 1C).

**Figure 1:**
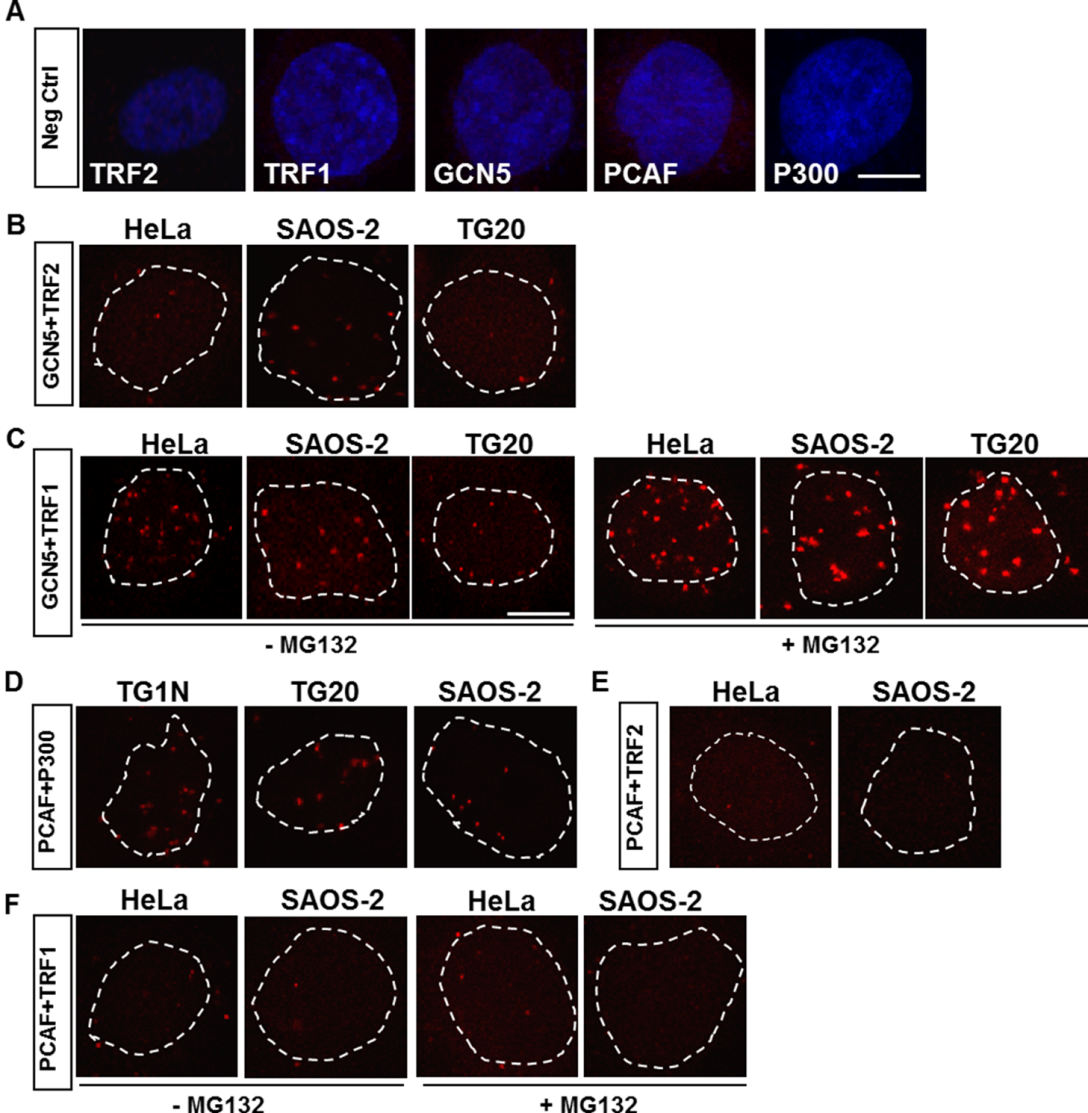
*In situ* proximity ligation assays (PLAs) for detection of protein-protein interactions (**A**) Representative PLA results for negative controls using only one of the primary antibodies against TRF2, TRF1, GCN5, PCAF or P300. The nuclei were stained with DAPI (blue). (**B**–**F**) Representative PLA results for telomerase-positive (TG1N and HeLa) and ALT (SAOS-2 and TG20) cell lines, using different combinations of primary antibodies. An interaction or close proximity between two proteins is demonstrated by a red spot resulting from a rolling circular amplification (RCA) reaction performed using labelled oligonucleotides. Nuclei are outlined with dashed white lines. In C and F, cells were treated or not with a proteasome inhibitor, MG132, at 1.5 h prior to fixation. Scale bar = 10 μm.

Immunostaining of GCN5 resulted in diffuse nuclear staining, and that of PCAF led to the formation of very large nuclear foci, preventing the investigation of their localization at telomeres (Supplementary Figure 1D–1E). We therefore performed *in situ* Proximity Ligation Assay (PLA, [18, 19]) to search for potential interactions between GCN5 and PCAF and the telomeric proteins TRF1 and TRF2 (Figure 1).

*In situ* PLA enables the detection of interactions of two distinct proteins by using primary antibodies raised from different species and species-specific secondary antibodies coupled to oligonucleotides. When the two proteins are in close proximity, the oligonucleotides from the secondary antibodies form a circular DNA molecule that can be amplified by rolling circular amplification (RCA) and visualized by the fluorescent labelling of oligonucleotides [18, 19].

PLA signals were observed using the anti-GCN5 and anti-TRF2 antibodies (Figure 1B) and were found to be present at a higher frequency using the anti-GCN5 and anti-TRF1 antibodies in both ALT and telomerase-positive cells (Figure 1C), which is consistent with a previous report showing that GCN5 interacts with telomeric proteins [5]. Consistent with the well-known association of these two lysine acetyltransferases [6], PLA revealed many interactions of PCAF with P300 (Figure 1D). However, no PLA signals were detected using the anti-PCAF and anti-TRF2 (Figure 1E) or the anti-TRF1 (Figure 1F) antibodies in ALT or non-ALT cells.

Proteasome inhibition has been shown to increase the interactions between TRF1 and GCN5 in telomerase-positive cells [5]. Consistently, we showed that treatment with a proteasome inhibitor, MG132, dramatically increased the number and intensity of PLA signals using GCN5 and TRF1 antibodies in both ALT and non-ALT cells (Figure 1C). However, this treatment did not promote any interactions between PCAF and TRF1 (Figure 1F).

These results confirmed the interactions of GCN5 with telomeric proteins in ALT cells as in telomerase-positive cells, contrary to PCAF that does not directly interact with telomeres.

### Opposite effects of GCN5 and PCAF knockdown on T-SCE in ALT cells

**Figure 2:**
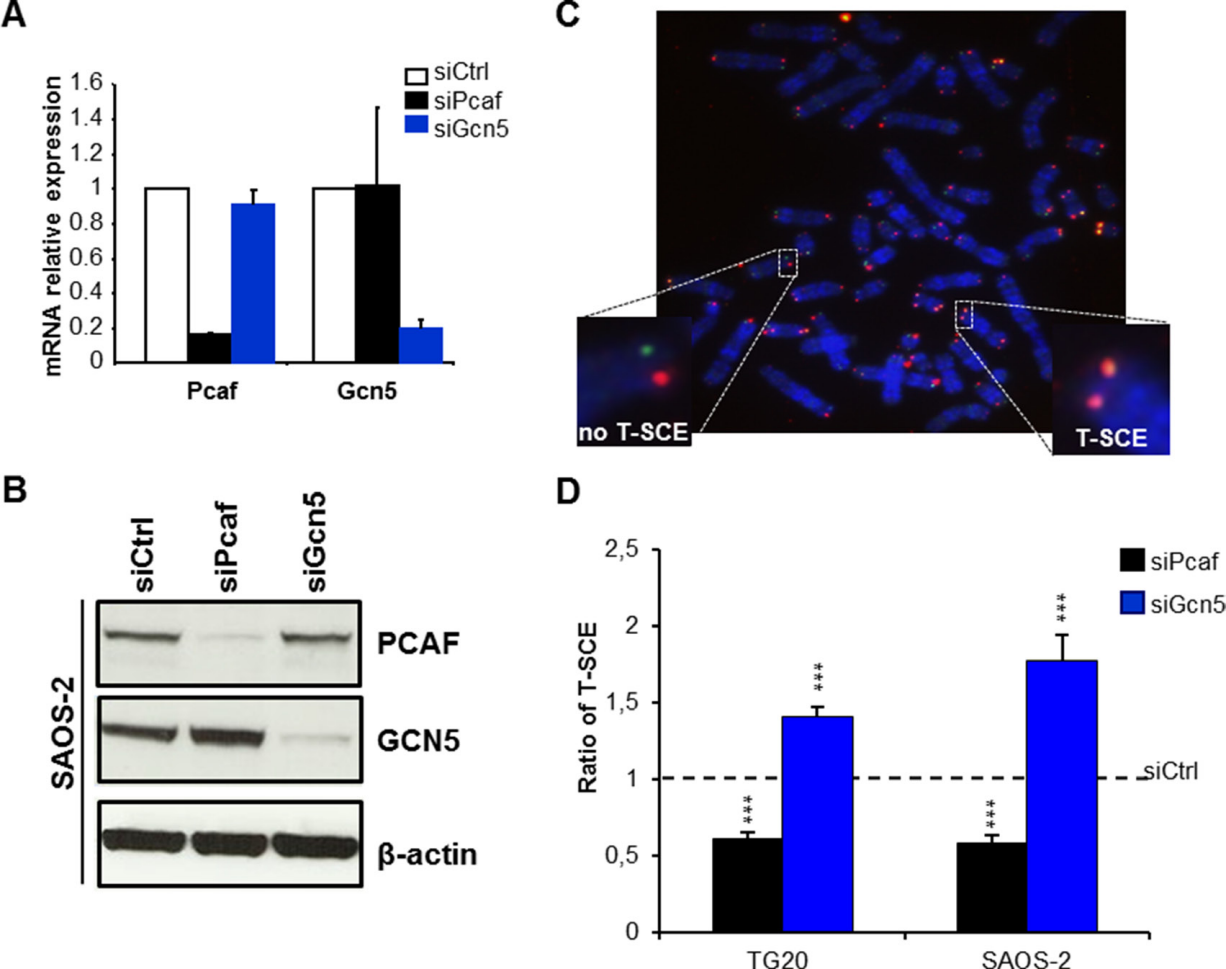
Opposite effects of PCAF and GCN5 down-regulation on telomere sister chromatid exchanges (T-SCEs) in ALT cells (**A**) mRNA expression levels of PCAF and GCN5 in TG20 cells transfected with siPcaf or siGcn5 relative to their expression in cells transfected with siCtrl, demonstrating the efficiency and specificity of the siRNAs. The error bars are the SEM from two independent experiments performed in duplicate. (**B**) Protein expression of PCAF and GCN5 in SAOS-2 cells transfected with siCtrl, siPcaf or siGcn5, also demonstrating the efficiency and specificity of the siRNAs. (**C**) Representative metaphase of SAOS-2 cells labelled using the CO-FISH technique. Successive hybridizations with an FITC-labelled (TTAGGG)_3_ PNA probe (green) and then with a Cy-3-labeled (CCCTAA)_3_ PNA probe (red) allowed for detection of the parental telomere C and G strands, respectively, by fluorescence microscopy. Yellow-stained telomeres were scored as T-SCE events. (**D**) T-SCE ratio after PCAF or GCN5 down-regulation. T-SCE ratios in TG20 and SAOS-2 cells transfected with siCtrl and siPcaf or siGcn5. The values are the ratio of T-SCE events (+SEM) relative to siCtrl for each cell line (ns=not significant, ***p < 0.001, as determined by Student's *t-test*). Between 2000 and 3500 chromosome extremities were analysed.

To investigate the importance of GCN5 and PCAF in ALT cells, we knocked down their expression using specific siRNAs (siGcn5 and siPcaf, respectively). As shown in Figure 2A, both GCN5 and PCAF were down-regulated at the RNA level by more than 80% compared to their expression in cells transfected with siCtrl at 48 h after transfection. We found that siGcn5 did not alter the *pcaf* mRNA level and that siPcaf did not affect the *gcn5* mRNA level (Figure 2A), ruling out both cross-reactions of the siRNAs due to the high level of homology between *gcn5* and *pcaf* and transcriptional cross-regulation between these two lysine acetyltransferases. The siRNA-mediated down-regulation of these mRNAs was further confirmed at the protein level (Figure 2B).

Inhibition of *gcn5* or *pcaf* expression did not have any significant short-term effect on cell viability or cell cycle progression in SAOS-2 ALT cells or HeLa cells (Supplementary Figure 2A–2D).

ALT-mediated telomeric elongation is related to frequent HR events, commonly referred to as T-SCEs [20]. We thus assessed whether GCN5 and PCAF knockdowns interfere with T-SCEs by performing CO–FISH on metaphase chromosomes [21, 22].

As previously described, T-SCEs are detected at high frequencies in metaphase spreads of ALT cells [20], such as TG20 and SAOS-2 cells (Figure 2C), but they are rare or absent in non-ALT cells, such as HeLa cells (Supplementary Figure 3A–3B). As shown in Figure 2D, GCN5 knockdown increased the frequency of T-SCEs by 1.41 ± 0.06-fold in the TG20 cells and by 1.77 ± 0.16-fold in the SAOS-2 cells (Figure 2D). By contrast, PCAF knockdown significantly decreased the frequency of T-SCEs by 0.39 ± 0.04-fold (*p* < 0.001) in TG20 cells and by 0.42 ± 0.06-fold (*p* < 0.001) in SAOS-2 cells compared to that in the cells transfected with siCtrl (Figure 2D).

GCN5 knockdown had no effect on the frequency of T-SCEs in telomerase-positive HeLa cells (Supplementary Figure 3B), demonstrating that the increase in T-SCEs caused by GCN5 knockdown was specific to ALT cells.

These data suggest thus that GCN5 may down-regulate telomere recombination in ALT cells, contrasting with PCAF, which may favour them.

### PCAF knockdown or GCN5 over-expression decreases the number of APBs in ALT cells

**Figure 3:**
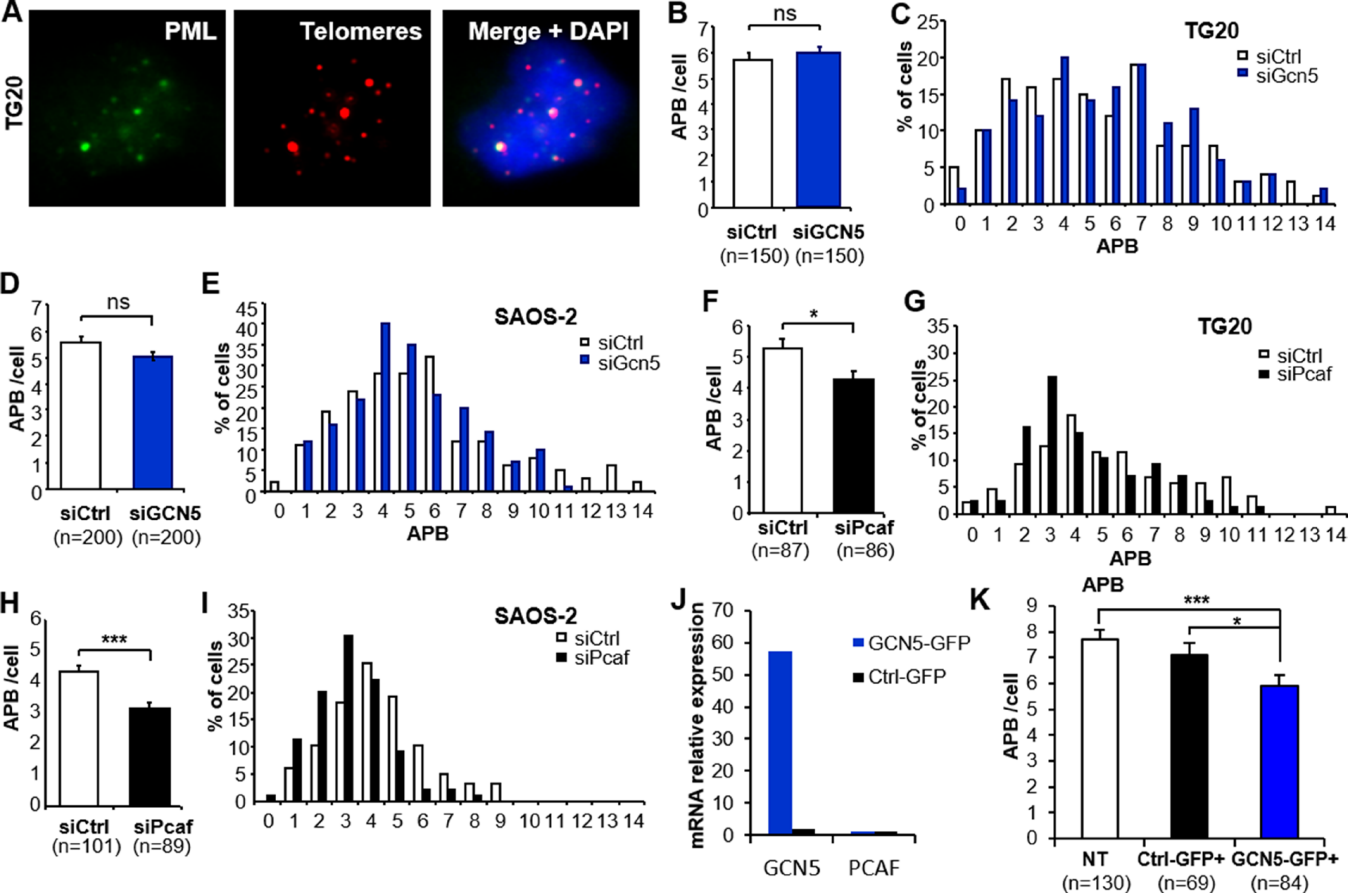
Down-regulation of PCAF or overexpression of GCN5 impairs ALT-associated PML body (APB) formation (**A**) Representative staining of APBs. One APB is scored when one PML focus (green) co-localizes with one red-stained telomere (Cy-3-labeled (CCCTAA)_3_ PNA probe). The images are reconstructed Z-stacks captured with confocal microscopy. (**B**–**I**) APB staining after GCN5 or PCAF down-regulation. APBs were scored in TG20 (B-C and F-G) and SAOS-2 (D-E and H-I) cells at 48 h after transfection with siCtrl and siGcn5 (B–E) or siCtrl and siPcaf (F-I). “n” indicates the number of counted cells. The values in B, D, F and H represent the average number of APBs per cell (+SEM). C, E, G and I show the distribution of transfected cells according to the number of APBs. (**J**–**K**) APB scoring after GCN5 overexpression in SAOS-2 cells. GCN5 and PCAF mRNA levels were determined by qRT-PCR in GCN5-GFP or Ctrl-GFP transfected SAOS-2 cells (J). APBs were scored as in A in non-transfected cells (NT) or cells transfected with control plasmid (Ctrl-GFP+) or GCN5 expessing plasmid (GCN5-GFP+). “n” indicates the number of counted cells. The values in B, D, F, H and K represent the average number of APBs per cell (+SEM). (ns = not significant, *p < 0.05, **p < 0.01, and ***p < 0.001, as determined by Student's *t-test*).

We next investigated the effects of the knockdown of these genes on another hallmark of ALT cells, APBs, which are PML bodies in which telomeres are elongated, that are present in ALT cells and absent in non-ALT cells [23]. The co-localization of PML bodies with telomeres was scored at 48 h after transfection with siCtrl, siGcn5 or siPcaf (Figure 3A).

GCN5 depletion did not induce a significant change in the number of APBs in TG20 cells (5.93 ± 0.28 and 5.71 ± 0.3 for siGcn5 and siCtrl, respectively, *p* = 0.59, Figure 3B–3C) or SAOS-2 cells (5.05 ± 0.17 and 5.56 ± 0.23 for siGcn5 and siCtrl, respectively, *p* = 0.07, Figure 3D–3E). In contrast, PCAF knockdown decreased the average numbers of APBs in both the TG20 cells (4.29 ± 0.25/cell and 5.3 ± 0.31/cell for siPcaf and siCtrl, respectively, *p* = 0.012, Figure 3F–3G) and SAOS-2 cells (3.1 ± 0.15/cell and 4.33 ± 0.18/cell for siPcaf and siCtrl, respectively, *p* < 0.001, Figure 3H–3I). We then tested whether over-expressing GCN5 in SAOS-2 ALT cells can affect APB formation. We found a decrease in the average number of APBs in cells overexpressing GCN5 48h after transfection (5.89 ± 0.40 and 7.11 ± 0.44 for GCN5-GFP and Ctrl-GFP transfected SAOS-2 cells, respectively, *p* = 0.0173, Figure 3J–3K and Supplementary Figure 4).

Thus, our data showed that the increase in T-SCEs was not associated with an increase in the number of APBs in GCN5 knockdown cells, however, overexpressing GCN5 altered APB formation in ALT cells. On the other hand, the decrease in T-SCEs in PCAF knockdown cells seems to be associated with a decrease in APBs. This last finding is consistent with a previous report showing that inactivation of the ALT mechanism is associated with the suppression of APB formation [23, 24].

P300, an interactor of PCAF, can bind to and acetylate the telomeric protein TRF2 [30]. To test whether P300 mediates the effect of PCAF on APBs, we scored the number of APBs in the absence of P300, PCAF or both P300 and PCAF. We found that depleting P300 alone reduced the APB formation in SAOS-2 and TG20 ALT cells but did not further decrease the number of APBs in combined absence of PCAF (Supplementary Figure 5A, 5C and 5F). The efficiency of siRNAs and transcripts of PCAF and P300 were verified in all conditions by qRT-PCR (Supplementary Figure 5B, 5D–E, 5G–H). These results indicate that PCAF and P300 may be cooperating in regulating APB formation.

### Opposite effects of GCN5 and PCAF knockdown on telomere instability in ALT cells

We assessed whether GCN5 or PCAF knockdown had an effect on telomere stability. We performed Telo-FISH on metaphase cells obtained at 48 h after transfection as previously described [22, 25].

Telomeres of ALT cells have been shown to display an “intermediate state” of capping, in which the telomeres are less saturated with shelterin, thereby inducing a DNA-damage response but still inhibiting end-to-end fusions [26]. Dicentric chromosomes are rare in metaphases of TG20 and SAOS-2 cells. Interestingly, they were not induced neither after PCAF knockdown nor after GCN5 knockdown, suggesting that neither PCAF nor GCN5 knockdown altered the function of shelterin in protecting telomeres from fusions.

**Figure 4:**
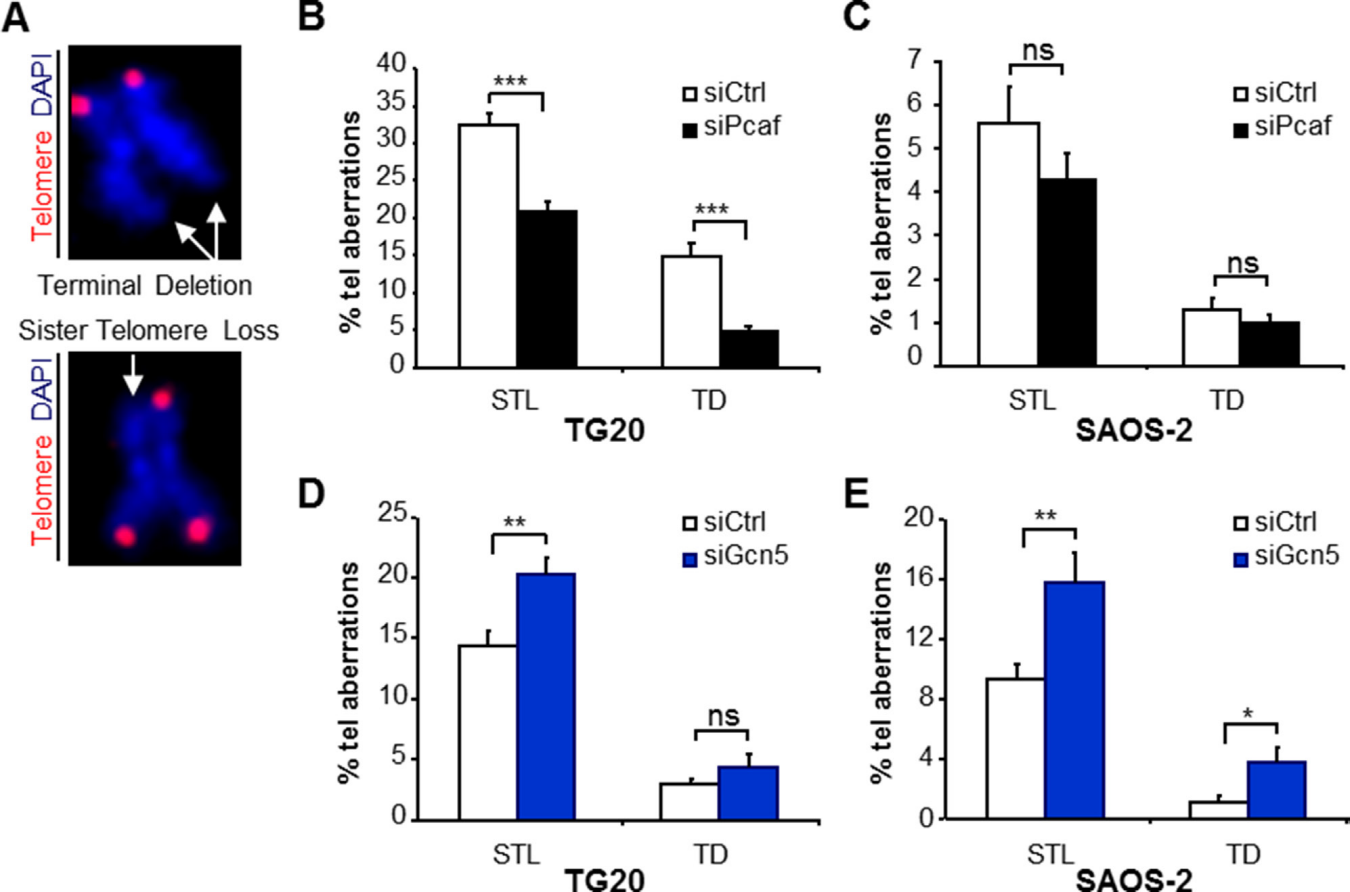
Opposite effects of PCAF and GCN5 down-regulation on telomere instability in TG20 GSCs and SAOS-2 cells (**A**) Telomere staining (red, Cy-3-labeled (CCCTAA)_3_ PNA probe) of metaphase chromosomes (blue, DAPI), showing the two main types of telomere aberrations (white arrows) found in metaphase spreads of TG20 and SAOS-2 with siCtrl, siPcaf or siGcn5: terminal deletion (TD) (upper panel) and sister telomere loss (STL) (lower panel). (**B**–**E**) STL and TD frequencies (+SEM) in TG20 and SAOS-2 cells transfected with siCtrl, siPcaf (B and C) or siGcn5 (D and E). The total numbers of analysed extremities were *n* = 1914, *n* = 2094, *n* = 2876 and *n* = 2788 for siCtrl and siPcaf in B and C, respectively, and *n* = 1882, *n* = 1702, *n* = 2044 and *n* = 1794 for siCtrl and siGcn5 in D and E, respectively.

As previously reported [17, 24], metaphases of ALT cells had a high number of telomere aberrations in which one or both sister chromatids lacked telomeric sequences, referred to as sister telomere losses and terminal deletions, respectively, showing a high telomere instability (Figure 4A). Interestingly, PCAF and GCN5 knockdown had opposite effects on these aberrations. While PCAF knockdown decreased sister telomere losses and terminal deletions in TG20 (Figure 4B) and SAOS-2 ALT cells (Figure 4C), GCN5 knockdown increased the frequencies of these telomere aberrations (Figure 4D–4E).

As these telomere aberrations increased or decreased very rapidly after GCN5 or PCAF knockdown, at the first or second metaphase after siRNA transfection, they are unlikely to be attributed to effects of these knockdowns on telomere erosion with cell divisions. Rather, changes in the frequencies of these aberrations likely reflect an increase or decrease in telomere instability allowing telomere recombination. Consistently, we showed that GCN5 knockdown increased telomere instability concomitant with an increase in T-SCEs, whereas PCAF knockdown decreased telomere instability concomitant with decreases in APBs and telomere recombination in ALT cells.

### Down-regulation of USP22 increased T-SCEs in ALT cells but had no effect on APBs

**Figure 5:**
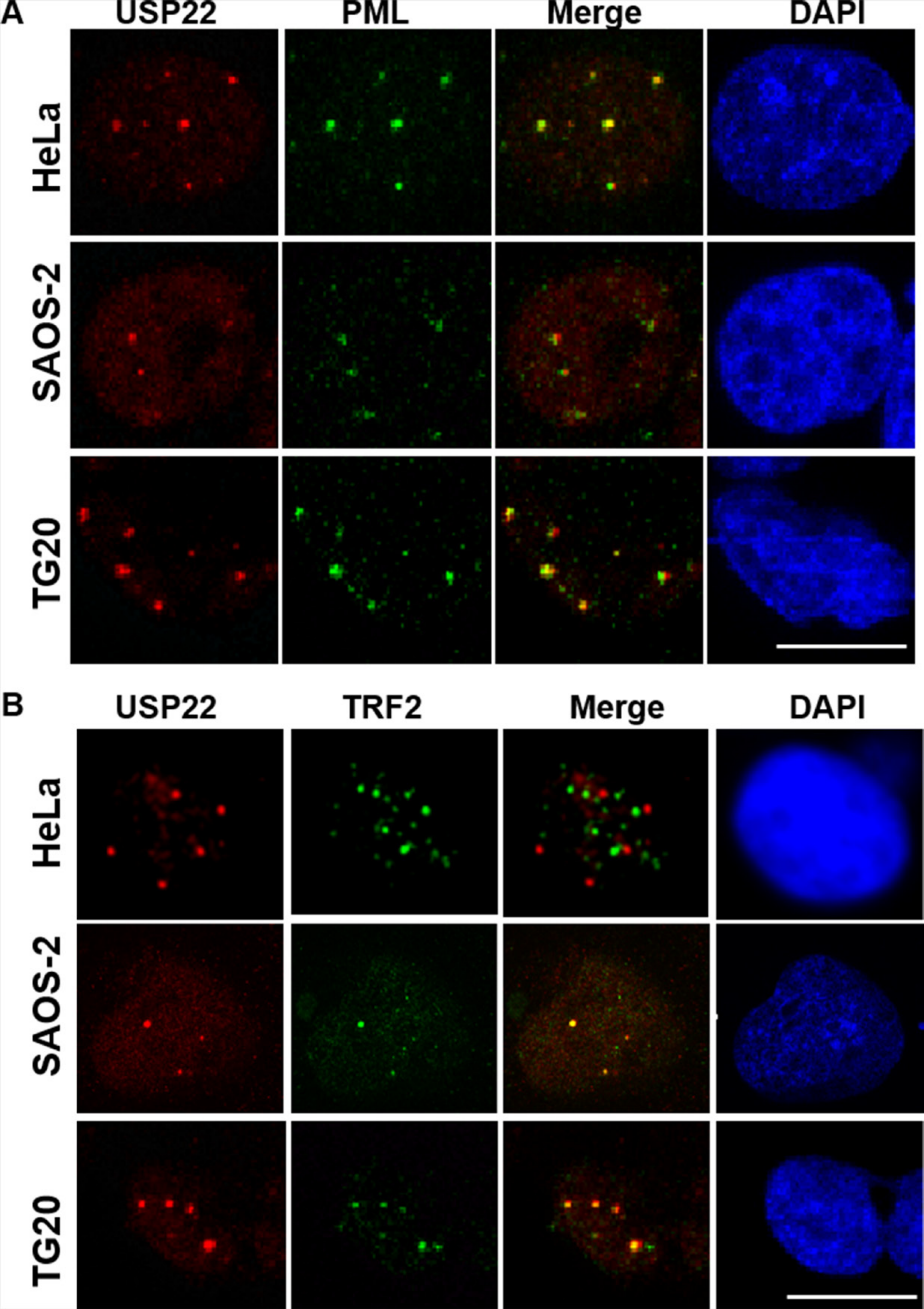
USP22 is a component of PML bodies and co-localizes with telomeric proteins in ALT cells (**A**) Immunofluorescence staining for USP22 (red) and PML (green), showing the co-localization of both proteins in telomerase-positive HeLa cells and TG20 and SAOS-2 ALT cells. The nucleus is shown in blue by DAPI staining. (**B**) Immunofluorescence staining for USP22 (red) and TRF2 (green), showing their co-localization in SAOS-2 and TG20 ALT cells but not in HeLa telomerase-positive cells. Scale bar = 10 μm.

GCN5 has been reported to control TRF1 turnover via its deubiquitination by ubiquitin-specific protease 22 (USP22) [5]. Interestingly, immunostaining revealed that USP22 formed large nuclear foci that co-localized with PML bodies in telomerase-positive cells as well as in ALT cells, suggesting that USP22 is a constitutive component of PML bodies (Figure 5A). Furthermore, we showed that USP22 also co-localized with large TRF2 foci detected in ALT cells but not in HeLa telomerase-positive cells (Figure 5B), demonstrating that USP22 is also present in APBs.

**Figure 6:**
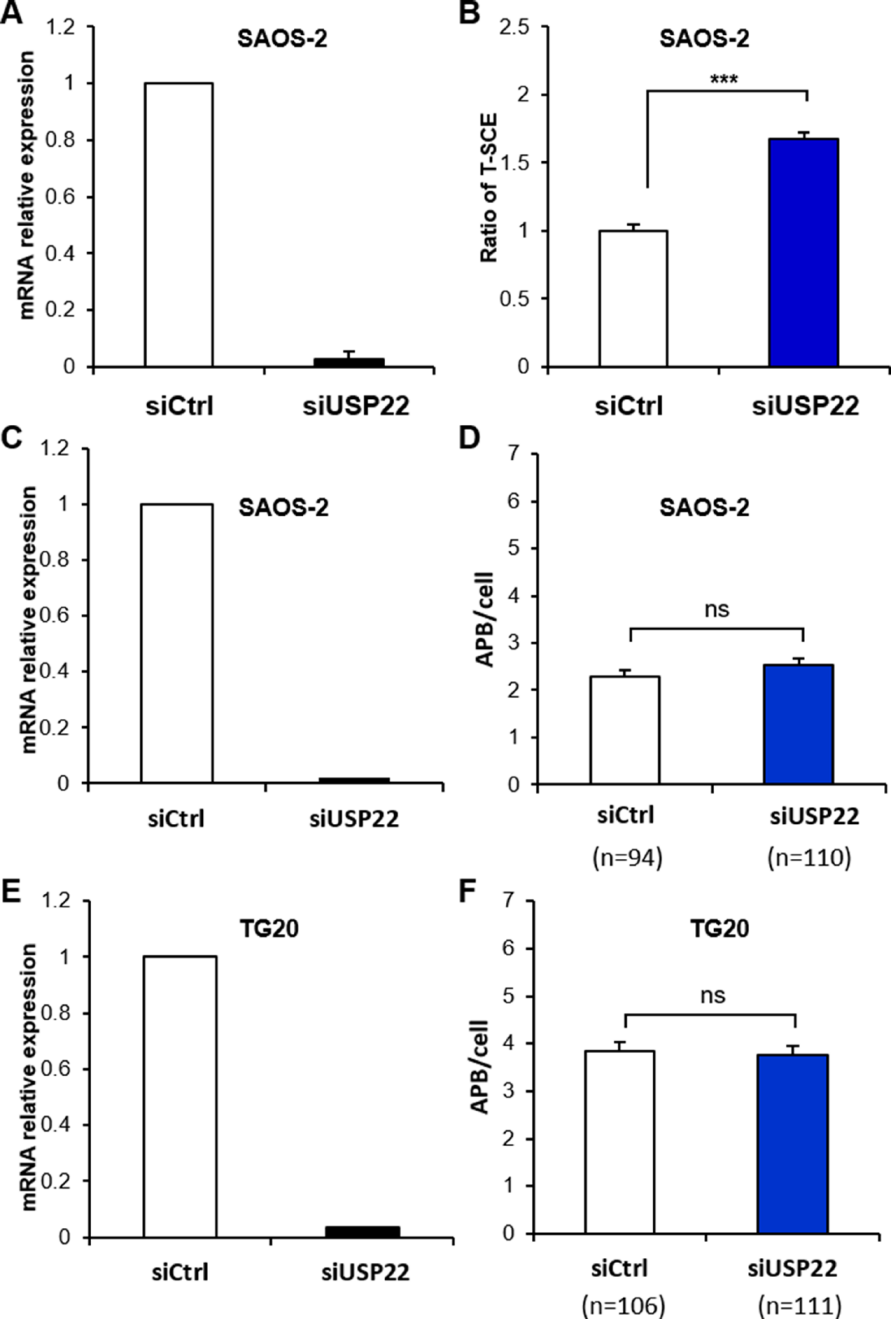
Down-regulation of USP22 increases T-SCEs in ALT cells but has no effect on APB formation (**A**, **C** and **E**) mRNA expression level of USP22 in SAOS-2 or TG20 cells transfected with siCtrl or siUsp22, demonstrating the efficiency of the siRNAs. The error bars are the SEM from two independent experiments performed in duplicate. (**B**) T-SCE scoring after USP22 down-regulation in SAOS-2 cells. The values are the ratio of T-SCE events (+SEM) relative to siCtrl for each cell line. (***p < 0.001, as determined by Student's *t-test*). (**D** and **F**) APB scoring after USP22 down-regulation. APBs were scored in SAOS-2 (D) and TG20 (F) cells at 48 h after transfection with siCtrl and siUSP22. “n” indicates the number of counted cells. The values represent the average number of APBs per cell (+SEM). (ns = not significant, as determined by Student's *t-test*).

Therefore, we transfected SAOS-2 cells with siUsp22, which resulted in the downregulation of USP22 mRNA by more than 97% after 48 h (Figure 6A). Interestingly we showed that USP22 knockdown increased T-SCEs by more than 1.5-fold compared with cells treated with siCtrl (Figure 6B) but had no effect on APB formation in both SAOS-2 and TG20 cells (Figure 6D–6F). USP22 knockdown had thus the same effect on ALT characteristics as GCN5 knockdown, suggesting that GCN5 may downregulate telomere recombination in ALT cells through its interaction with USP22.

## DISCUSSION

GCN5 and PCAF are known to have different roles and targets, functioning in large multi-subunit complexes, such as SAGA-like complexes, in a mutually exclusive manner [7–11]. Here we show in two ALT cell lines that GCN5 knockdown increased T-SCE and telomere instability, whereas PCAF knockdown decreased T-SCE, APBs formation and telomere instability in ALT cells. GCN5 and PCAF knockdowns had thus differential effects on ALT, up-regulating it or down-regulating it respectively. These data support thus the hypothesis that these homologous lysine actetyl-transferases may have a role in the regulation of this mechanism of telomere maintenance.

We have shown that GCN5 interacts with TRF1 in ALT cells, as previously reported in telomerase-positive cells [5]. The TRF1 steady state is tightly regulated by post-translational modifications such as ubiquitination [3]. According to Atanassov et al. [5], GCN5 contributes to telomere stability in telomerase-positive cells by regulating TRF1 turnover via interacting with USP22. GCN5 has been shown to support the association of USP22 with the SAGA complex. This association allows USP22 to deubiquitinate TRF1 and to promote the binding of TRF1 to telomeres rather than undergoing ubiquitin-mediated proteolysis [5]. We have reported for the first time that USP22 is present in PML bodies and APBs in ALT cells. Interestingly, USP22 knockdown increases T-SCEs in ALT cells but has no effect on APB and has thus similar effects to GCN5 knockdown. These findings support the hypothesis that GCN5 and USP22 act together in APBs to oppose the ALT mechanism.

TRF1 has been shown to control the association of POT1, another telomeric protein, with single-stranded telomeric DNA [27]. When present at telomeres, POT1 prevents the activation of ATR at telomeres [28], which we have recently demonstrated is critical for ALT cells [29]. This raises the hypothesis that once USP22 associates with SAGA and GCN5, it may oppose telomere recombination in APBs by promoting the binding of POT1 to telomeres via TRF1, thereby inhibiting ATR activation. Importantly, GCN5 knockdown did not increase T-SCEs in HeLa cells, showing that the downregulation of GCN5 is not able to induce the ALT mechanism in telomerase-positive cells.

In contrast with GCN5 and P300 [30], PCAF has not been shown to participate in telomere maintenance. We found that PCAF did not interact directly with telomeric proteins in ALT cells, similarly as a previous report showing that PCAF is not present at telomeres in telomerase-positive cells [30]. These findings suggest that the effects of PCAF knockdown on ALT are indirect. We have shown that both siPcaf and siP300 decreased the number of APBs suggesting that they are involved together in APBs formation. P300 acetylates TRF2, stabilizing telomeres [30]. Further experiments are required to determine whether P300-mediated TRF2 acetylation is involved in APBs formation in ALT cells.

GCN5- and PCAF complexes share many common subunits [7–11], suggesting that GCN5 and PCAF may compete for interactions with these subunits. Knockdown of PCAF could thus increase the formation of GCN5-complexes, thereby increasing the activities of GCN5/USP22 at telomeres and, in the end, down-regulating ALT. This hypothesis is sustained by the overexpression of GCN5, showing a down regulation of the ALT mechanism as well.

Our data raise the hypothesis that the relative expressions of GCN5 and PCAF may be involved in ALT regulation. In this model, PCAF would increase ALT by competing with GCN5 for interactions with common partners, which prevents the stabilizing activities of GCN5/USP22 at telomeres. Although further studies are required to challenge this hypothesis and investigate other putative roles of GCN5 and PCAF, for instance at the transcriptional level, our data suggest that stimulating GCN5/USP22 activity can oppose to maintenance of telomeres in ALT cells and thus could be considered for the development of new therapeutic strategies in ALT cancers.

## MATERIALS AND METHODS

### Cell culture

Human glioma stem-like cells (GSCs) were derived from tumour samples and grown as previously described [16, 17, 31]. Telomerase-positive HeLa cells and SAOS-2 osteosarcoma ALT cells were also grown as previously described [16].

### Quantitative real-time PCR (qPCR)

RNA was extracted using an RNeasy Plus Mini Kit (Qiagen) according to the manufacturer's instructions. Isolated RNA was transcribed to cDNA using High Capacity RNA-to-cDNA Master Mix (Applied Biosystems). Quantitative PCR reactions were performed in 96-well plates in duplicate using SYBR Green Master Mix (Applied Biosystems) and the following primers: *gapdh* primers: forward: 5′-GTCGCCAGCCGAGCCACATC-3′, reverse: 5′-GGTGACCAGGCGCCCAATACG-3′; *pcaf* primers: forward: 5′-GCCACAGTTCTGCGACAGTCT-3′, reverse: 5′-CCGAGCGAAGCAATGTTCTC-3′; *gcn5* primers: forward: 5′-GTGCTGTCACCTCGAATGAG-3′, reverse: 5′-TGGAGAAACCCTGCTTTTTGA-3′; *p300* primers: forward: 5′CGCTTTGTCTACACCTGCAA-3′, reverse: 5′-TGCTGGTTGTTGCTCTCATC-3′, and *usp22* primers, forward: 5′-CTCCTGTCTGGTCTGTGAGAT G-3′, reverse: 5′-CAGCAACTTATACGGGATGTGA-3′ (EuroGentec).

### siRNA transfection

The *pcaf, p300, usp22* and *gcn5* genes were knocked down using stealth siRNAs (Life Technologies). Cells were dissociated and transfected with a final concentration of 20 nM siRNAs targeting PCAF (siPcaf), P300 (siP300), USP22 (siUSP22) or GCN5 (siGcn5) or negative control siRNAs (siCtrl) by electroporation at 1050 V for 40 ms using a Neon Transfection System (Life Technologies) according to the manufacturer's instructions. Transfected cells were then plated on laminin-coated flasks (for chromosome orientation-fluorescence *in situ* hybridization (CO-FISH) experiments), 96-well plates (for cell proliferation assays) or Millicell EZ slides (PEZGS0816, Millipore, for immunofluorescence (IF) assays). Knockdown efficiency was verified at 48 h after transfection by qPCR, western blotting or IF. The siRNA sequences were as follows:

siPcaf:

Sense sequence: 5′-CCACUUUAAUGGGAUGUG

AGCUAAA-3′,

Antisense sequence: 5′-UUUAGCUCACAUCCCA

UUAAAGUGG-3′;

siGcn5:

Sense sequence: 5′-CCAAGCAGGUCUAUUUCUA

CCUCUU-3′,

Antisense sequence: 5′-AAGAGGUAGAAAUAGA

CCUGCUUGG-3′;

siUsp22:

Sense sequence: 5′-GGAGAGAAGUUUUCAAC

UUtt-3′,

Antisense sequence: 5′-AAGUUGAAAACUUCUC

UCCaa-3′.

sip300-1:

Sense sequence: 5′-GGAUUCGUCUGUGAUGGC

UGUUUAA-3′

sip300-2:

Sense sequence: 5′-CAGGUAUGAUGAACAGUC

CAGUAAA-3′

### Transient over-expression of GCN5 in SAOS-2 cells

The plasmid pEGFP- GCN5 (here GCN5-GFP) and the control plasmid pEGFP (here Ctrl-GFP) have been constructed as follow: the HsGCN5-1-837 encoding sequence has been amplified from Addgene plasmid # 74784. The resulting PCR product was inserted by SLIC [33] in pEGFP-N1 plasmid digested by Acc65I-NheI. In order to express the gene of interest independently of the GFP, the stop codon was included in the amplicon and the pEGFP-N1 vector backbone was modified in order to add an internal ribosome entry site (IRES) sequence. Thus, a DNA fragment encompassing an internal ribosome entry site (IRES) followed by 3 NLS and the GFP encoding sequence obtained by amplifying the pCIG plasmid (a derivative of pIRES2-AcGFP obtained from G. Livera) was inserted by SLIC in the previously obtained plasmid digested by AgeI-BsrGI. As a control plasmid, the PCR product encompassing the IRES sequence, the 3 NLS and the GFP encoding sequence was inserted in pEGFP-N1 plasmid as just described above. The resulting plasmids were sequence verified. All enzymes were from New England Biolabs (Beverly, MA).

Primers for amplifying HsGCN5-1-837

SP0678: 5′ – GTTTAGTGAACCGTCAGATCCGC

TAGCATGGCGGAACCTTCC – 3′

SP0679: 5′ – CCGGTGGATCCCGGGCCCGCGG

TACCTACTTGTCAATGAGGCCTC – 3′

Primers for amplifying IRES-3NLS-GFP

SP0680: 5′ – GGGCCCGGGATCCACCGGTGCCC

CTCTCCCTCC – 3′SP0681: 5′ – GGCCGCTTTAC

TTGTACA – 3′

Annealing sequences on target sequence were underlined. Homology sequences with plasmid used for SLIC cloning were in italics. Restriction sites were bolded (*Nhe*I for SP0678, *Acc*65I/*Kpn*I for SP0679, *Age*I for SP0680 and *BsrG*I for SP0681). Transient transfection of DNA plasmids into SAOS-2 cells were performed using lipofectamine 3000 (Invitrogen) according to manufacturers’ instructions. Briefly, SAOS-2 cells were grown to 70–80% confluence in 6 well plates and a mix of plasmid DNA and lipofectamine was applied for 2 hours before changing the medium. Quantitative RT-PCR, immunofluorescence and detection of APBs were performed 48h post transfection as described elsewhere in Material and Methods.

### Western blot

Cell pellets were dried, and then cells were lysed in RIPA buffer containing protease and phosphatase inhibitors. The following antibodies were used for detection of the desired proteins: PCAF (1:1000, sc13124, Santa Cruz), anti-GCN5 (1:1000, #3305, Cell Signalling), anti-PML (1:1000, sc966, Santa Cruz), α-tubulin (1:1000, T6199, Sigma-Aldrich), and β-actin (1:1000, A1978, Sigma-Aldrich).

### Immunofluorescence

At 48 h after transfection with siRNAs, adherent cells were fixed by incubation for 10 min in 4% paraformaldehyde and were then permeabilized by incubation with 0.1% Triton X-100 in phosphate-buffered saline (PBS) for 10 min at room temperature. For blocking, cells were incubated for 1 h at room temperature in PBS containing 7.5% goat serum and 7.5% foetal bovine serum and then incubated overnight at 4°C with the indicated primary antibody. The following antibodies were used for IF: GFP (1/200, ab6673, abcam), PML (1:100, sc966, Santa Cruz), PCAF (1:50, ab12188, Abcam), GCN5 (1:100, 3305, Cell signaling), TRF2 (1:100, IMG-124A, Imgenex), and USP22 (ab4812, Abcam). Secondary labelling was performed using an Alexa Fluor 488- or 594-conjugated antibody (Molecular Probes) at room temperature for 1 h.

### WST-1 cell proliferation assay

Transfected cells were plated in 96-well plates. A total of 2,000 cells were used per well. WST-1 assay (11644807001, Roche) was then performed at 24 and 48 h following transfection according to the manufacturer's instructions.

### Cell cycle

Cell cycle analysis was performed using propidium iodide. Cells were trypsinized and fixed with cold ethanol (70°) for 10 min at −20°C and then incubated with propidium iodide (50 μg/mL) and RNase for 1 h at 37°C. The DNA concentrations in the stained samples were subsequently measured by flow cytometry using an LSRII cytometer (BD Bioscience), and cell cycle distribution was analysed using Flowjo software.

### T-SCE analysis

CO-FISH was performed as previously described [32]. Cells were cultured in complete medium supplemented with 10 μM BrdU for one cell cycle. Metaphase spreads were stained with Hoechst 33258, exposed to UV light and digested with exonuclease III (Promega). Successive hybridizations with an FITC-labelled (TTAGGG)_3_ PNA probe and then with a Cy-3-labelled (CCCTAA)_3_ PNA probe (Applied Biosystems) allowed for detection of the parental telomere C and G strands, respectively. Metaphases were captured and analysed using an Axio Imager Z.2 (Zeiss, Oberkochen, Germany) coupled to a Metafer Image Analysis System (MetaSystems, Altlussheim, Germany).

### Telomere staining

Telomere staining was performed by fixation for 2 min in 4% formaldehyde and dehydration with ethanol (50°-80°-100°) on ice. The dehydrated slides were then stained with a (CCCTAA)_3_ PNA probe (Applied Biosystems) in PNA hybridization solution (70% formamide, 10 mM Tris, pH 7.2, and 1% BSA) by denaturation at 80°C for 3 min and then hybridization at room temperature for 2 h. Next, the slides were washed with 70% formamide and 10 mM Tris, pH 7.2, and washed 3 times with 50 mM Tris, pH 7.2, 150 mM NaCl, and 0.05% Tween-20, counterstained with DAPI and mounted. Images of APBs were taken using a Leica DM 2500 microscope and analysed using LAS AF Lite Leica software. Metaphases were captured and analysed using an Axio Imager Z.2 (Zeiss, Germany) coupled to a Metafer Image Analysis System (MetaSystems, Germany).

### Proximity ligation assay (PLA)

PLA (Duolink II Fluorescence, Olink Bioscience) was used for the detection of protein-protein interactions according to the manufacturer's instructions. Briefly, adherent cells grown on round glass slides in 24-well plates were fixed for 10 min with 4% paraformaldehyde, permeabilized in PBS containing 0.5% Triton X-100 for 20 min at room temperature, and then blocked by incubation in PBS containing 7.5% goat serum and 7.5% foetal bovine serum for 1 h at room temperature. Next, the cells were incubated overnight at 4°C with two antibodies against the two proteins of interest. The following primary antibodies were used: TRF2 (1:100, IMG-124, Imgenex), PCAF (1:100, AB9962, Millipore), GCN5 (1:100, H-75, Santa Cruz), TRF1 (1:100, ab10579, Abcam), and P300 (1:100, NA 46, Calbiochem). The slides were then washed three times with 0.1% Triton X-100 in PBS and incubated with two PLA probes (PLA probe MINUS stock and PLA probe PLUS stock; Duolink II) for 1 h at room temperature. Subsequently, the slides were washed three times with 0.1% Triton X-100 in PBS. Ligation was conducted for 30 min at 37°C, followed by two washes with 0.1% PBS and Triton X-100 and amplification using DNA polymerase (Duolink II) for 100 min at 37°C. The slides were then washed, dried and mounted with Dapi-Fluoromount-G (Southern Biotech). Images were captured using a Leica DM 2500 microscope.

## SUPPLEMENTARY MATERIALS FIGURES


