# Supplementary material for: Opposite effects of GCN5 and PCAF knockdowns on the alternative mechanism of telomere maintenance
Source: Oncotarget. 2017 Feb 17;8(16):26269–80. doi: 10.18632/oncotarget.15447 (PMC5432255; doi:10.18632/oncotarget.15447)
Supplement: Supplementary file 1 [file oncotarget-08-26269-s001.pdf]

Opposite effects of GCN5 and PCAF knockdowns on the alternative mechanism of telomere maintenance

Supplementary Materials

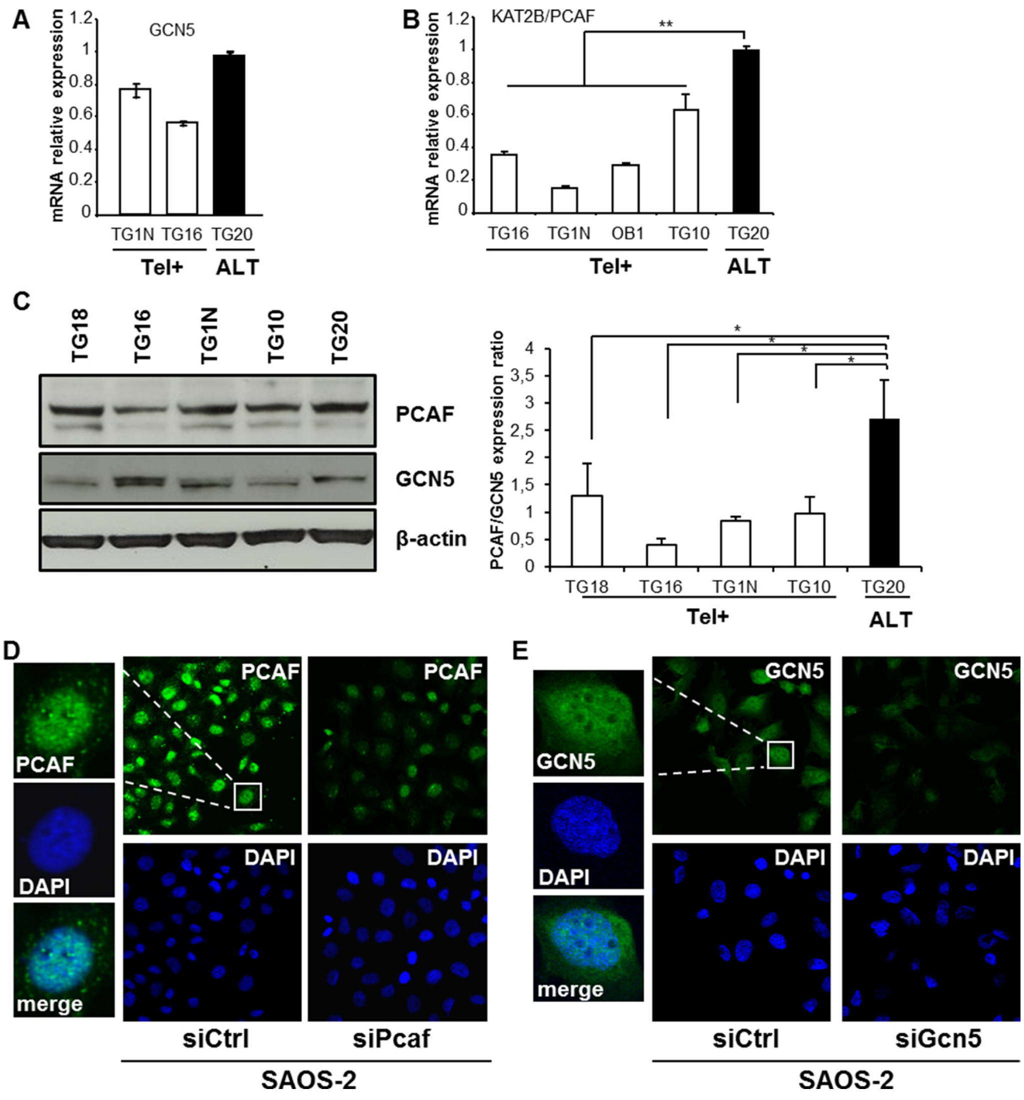

Supplementary Figure 1: Expression profiles of PCAF and GCN5 in used cell lines. (**A–B**) Expression profiles of GCN5 and PCAF, as determined by qRT-PCR. mRNA expression levels of GCN5 (A) in telomerase-positive GSCs (TG1N and TG16) and of KAT2B/PCAF (B) in telomerase-positive GSCs (TG16, TG1N, OB1, and TG10) relative to their expression in TG20 ALT GSCs. The error bars are + SEM from at least two independent experiments performed in duplicate. (**$p < 0.01$, as determined by Student's *t*-test). (**C**) PCAF and GCN5 protein expression, as determined by Western blotting, in GSCs. The graph shows the expression ratio of PCAF relative to GCN5 after normalization by actin as a loading control. The values (+SD) were calculated from three independent experiments (*$p < 0.05$ as determined by the Mann-Whitney test). (**D**) Immunostaining for PCAF (green) in SAOS-2 cells transfected with siCtrl or siPcaf, showing PCAF down-regulation. (**E**) Immunostaining for GCN5 (green) in SAOS-2 cells transfected with siCtrl or siGcn5, showing GCN5 down-regulation. The left panels in D and E depict a magnification of one cell. The nucleus is stained with DAPI (blue).

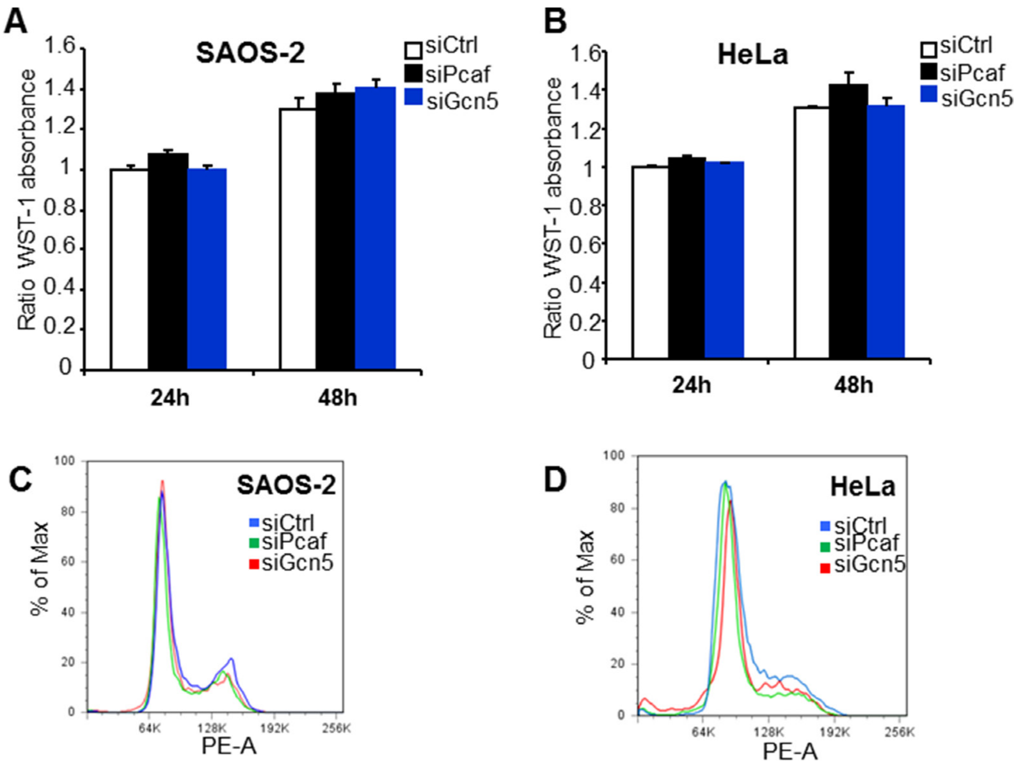

	siCtrl	siPcaf	siGcn5
%G1	64.48	68.94	70.81
%S	11.53	11.49	10.39
%G2/M	22.34	18.03	16.06

	siCtrl	siPcaf	siGcn5
%G1	74.19	79.18	69.27
%S	10.8	8.25	11.68
%G2/M	13.02	10.06	12.35

Supplementary Figure 2: Effects of PCAF and GCN5 down-regulation on proliferation and the cell cycle. (**A**–**B**) WST-1 proliferation assays of SAOS-2 and HeLa cells at 24 and 48 h after transfection with siCtrl, siPcaf or siGcn5. The values were calculated relative to WST-1 absorbance in cells transfected with siCtrl at 24 h. The error bars are the SEM from 4 replicates. (**C**–**D**) Cell cycle distributions of SAOS-2 and HeLa cells transfected with siCtrl, siPcaf or siGcn5 at 48 h after transfection. The lower panels show the percentages of cells in each cell cycle phase (G1, S and G2/M) for each condition.

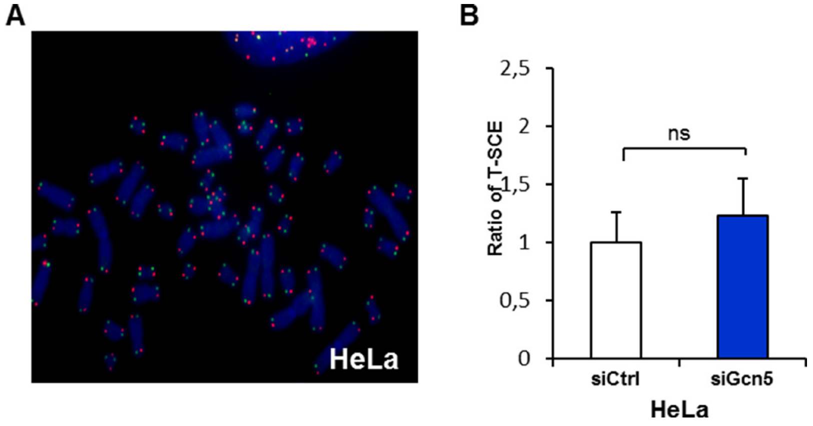

Supplementary Figure 3: T-SCEs in HeLa cells after down-regulation of GCN5. (**A**) Representative metaphase HeLa cells labelled with the CO-FISH technique. Successive hybridizations with a FITC-labelled $(TTAGGG)_3$ PNA probe (green) and then with a Cy-3-labelled $(CCCTAA)_3$ PNA probe (red) allowed for detection of the parental telomere C and G strands, respectively, by fluorescence microscopy. No yellow-stained telomeres corresponding to T-SCE events were observed. (**B**) The T-SCE ratio after GCN5 down-regulation. The T-SCE ratios in HeLa cells transfected with siCtrl and siGcn5. The values are the ratio of T-SCE events (+SEM) relative to siCtrl. (ns = not significant, as determined by Student's *t*-test).

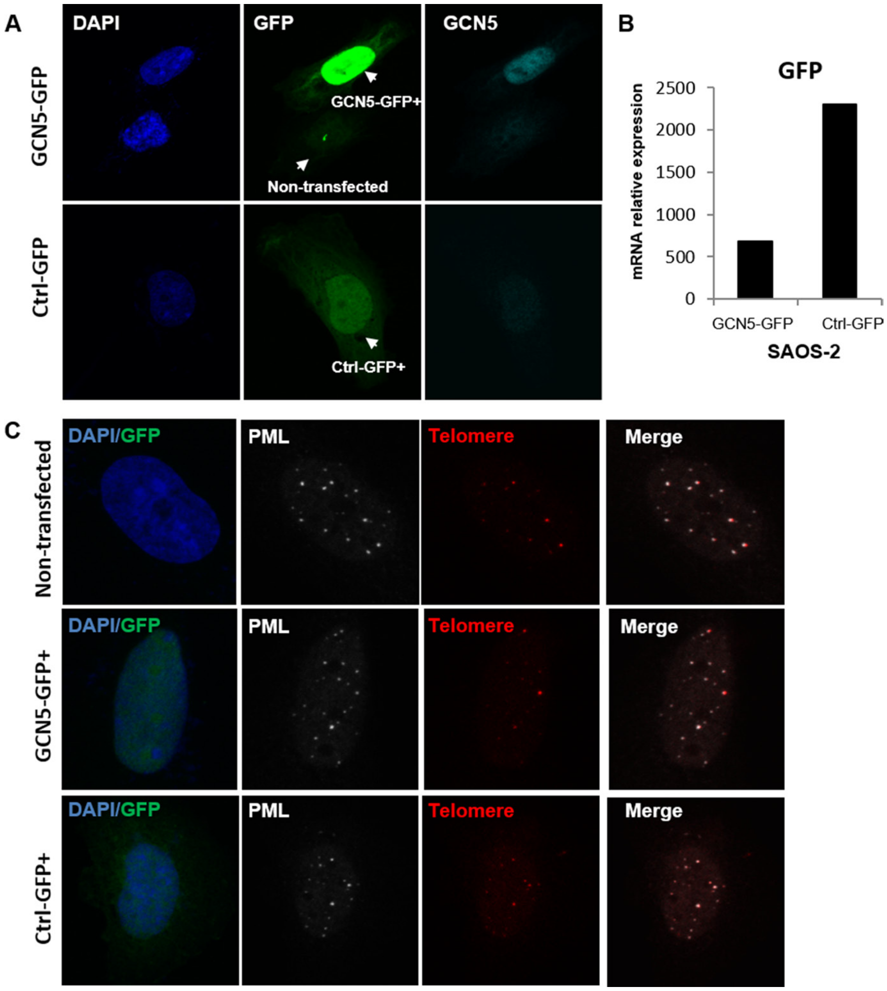

Supplementary Figure 4: APB scoring in GCN5 over-expressing SAOS-2 cells. (**A**) Representative images showing GFP and GCN5 staining in GCN5-GFP transfected cells (GCN5-GFP+), control cells (Ctrl-GFP+) or non-transfected cells. (**B**) qRT-PCR results showing GFP mRNA levels in SAOS-2 GCN5-GFP or Ctrl-GFP transfected cells. *eGFP* primers: forward: 5′-GCAAAGACCCCAACGAGAAG-3′, reverse: 5′-TCACGAACTCCAGCAGGACC-3′; (**C**) Representative images showing APB staining in cells from A. One APB is scored when one PML focus (white) co-localizes with one red-stained telomere (Cy-3-labeled $(CCCTAA)_3$ PNA probe). GFP staining (green) was used to label transfected cells.

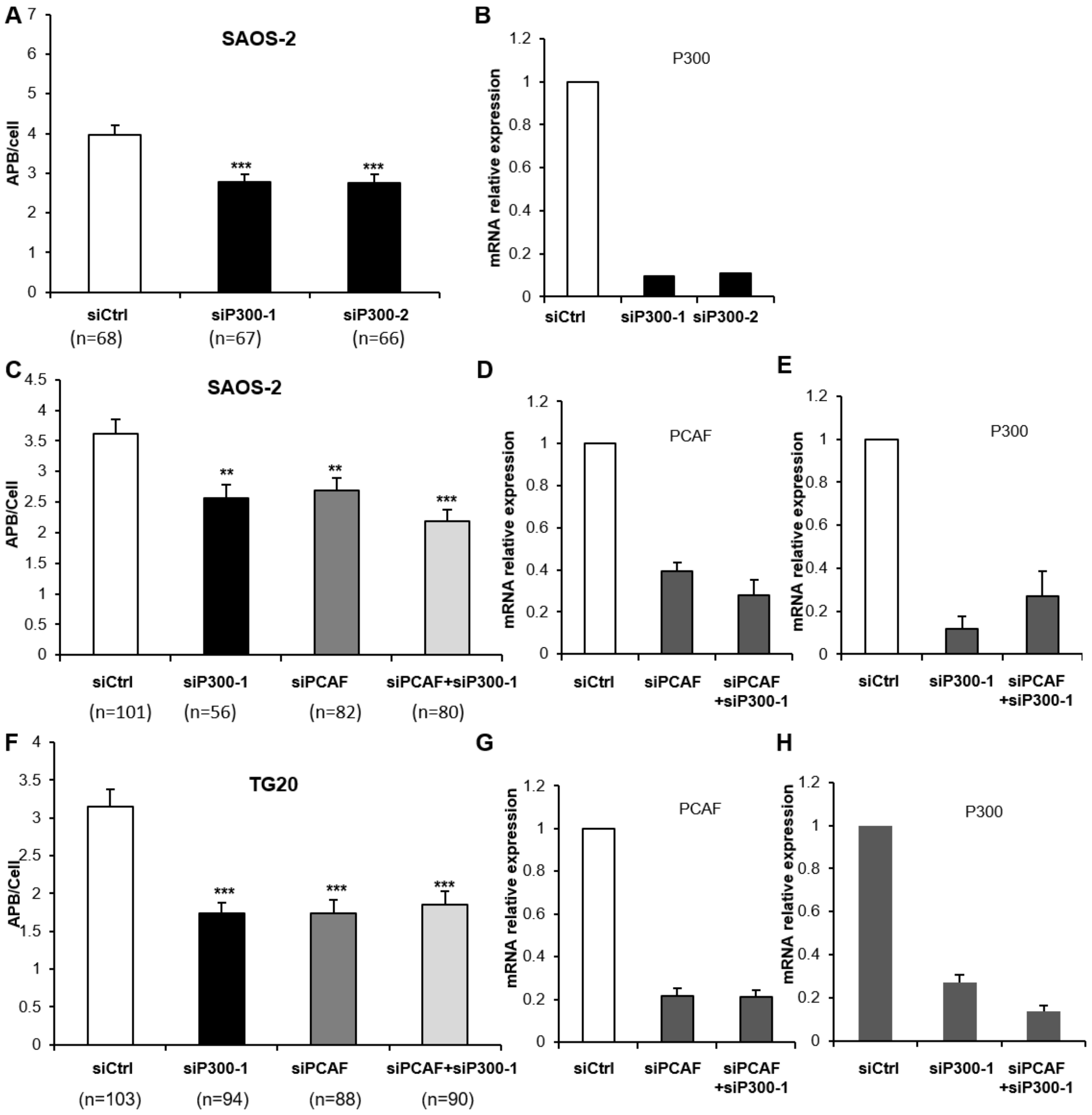

Supplementary Figure 5: APB scoring after P300 or P300 and PCAF down-regulation in ALT cells. (**A**, **C** and **F**) APBs were scored in SAOS-2 (A,C) and TG20 (F) cells at 48 h after transfection with siCtrl, siP300 (1 or 2 referring to two different siRNAs), siPCAF or siP300-1 +siPCAF. "n" indicates the number of counted cells. The values in the graph represent the average number of APBs per cell (+SEM). (***$p < 0.001$, **$p < 0.01$, as determined by Student's *t*-test). (**B**, **D–E**) and (**G–H**) qRT-PCR results showing mRNA expression levels of PCAF and P300 in cells used in A, C and F.
